# Gastric Dyspepsia

**Published:** 1892-02-06

**Authors:** 


### The Practitioner's Mirror and Retrospect.

[*The Editor will be glad to receive offers of co-operation and contributions from members of the profession. All letters should be addressed to* The Editor, The Lodge, Porchester Square, London, W.]

### MEDICINE.

### GASTRIC DYSPEPSIA.

Many valuable contributions have of late been made to our knowledge of the vexed question of dyspepsia. We are still hampered by the very prevalent idea that pepsin is the chief or only agent required for digestion in the stomach, the result being that pepsin or pancreatin is frequently administered without the anticipated relief to the patient being obtained. Now M. Georges has made a number of experiments on the relative digestive powers of natural and artificial gastric juices. Cubes of hard-boiled white of egg were placed for a certain length of time in an artificial gastric juice containing $HC_l$ and pepsin in varying dilution. He found that a combination of 40 cubic centimetres of a 0·4 per cent. solution of $HC_l$ with 8 to 10 centigrammes of pepsin, gave the best results of all. His observations on the digestive capabilities of natural gastric juice were made on 142 specimens taken from 69 patients. In eight cases of chlorotic anæmia the contents of the stomach were found on eighteen occasions to have no digestive power at all; on two occasions they possessed a slight power; and on one occasion digestive power was normal. In each of these cases the addition of pepsin failed to modify the digestive action; in one instance, indeed, it appeared to delay it. The addition of $HC_l$, on the contrary, had a very marked effect in twelve cases, but was without result in eight cases. Of all the degrees of dilution employed, 0·4 to 0·6 per cent. of $HC_l$ gave the best results. In nine cases of gastric ulcer the digestive power of the gastric juice was found to be excellent on nineteen occasions, and absent altogether on ten. In these ten instances pepsin was found to be hurtful in its action on three occasions, while $HC_l$ (0·4 per cent.) restored the digestive power on six occasions. Analogous results were obtained in 92 specimens of gastric juice obtained from 52 different cases, including such affections as tabes, catarrhal jaundice, acute and chronic gastritis. The result, therefore, of the observations on 142 samples of gastric juice was that 115 were found without digestive power. In all these cases pepsin had no action, appearing sometimes to be even hurtful. The conclusive he draws from these data seems then justified, namely, that pepsin is useless where the dyspepsia is due to some primary or secondary disturbances in the gastric secretion, while hydrochloric acid can be used with advantage in such cases. A further series of observations on the digestive power possessed by various medicinal preparations containing as their basis hydrochloric acid, pepsin, pancreatin, or papain, showed that they were without action in all cases; they did not facilitate digestion either in the case of artificial or of natural gastric juice.

There is no doubt then that the presence of a normal proportion of free hydrochloric acid is essential to good digestion. Von Jaksch found that free hydrochloric acid is present in considerable quantity as early as a quarter of an hour after the ingestion of food. The greatest quantity of acid found in any one case—·1615 per cent.—was obtained on a milk diet; slightly less on a meat diet, ·1563 per cent.; and least of all on a carbohydrate diet, ·1102 per cent. Digestion appears to be completed most rapidly on a meat diet, next in order coming carbohydrate and milk diet. He concludes that entire absence of free hydrochloric acid from the gastric juice, or its presence in traces only, during the first quarter to half an hour of digestion, is without pathological significance. If no free acid is present one to three hours after ingestion of meat or milk, it is extremely probable that some severe disturbance of gastric function exists. On a carbohydrate diet, on the other hand, the presence of a mere trace of free hydrochloric acid is quite consistent with conditions of health. Furthermore, we are not justified in concluding that the presence of free acid in considerable quantity two to three hours after ingestion of meat or milk points to some pathological condition, as wide variations are possible within physiological limits.

Further, Dr. Leopold Wohlmann reports (*f. Kinderhlk.* Band xxxii., Heft 3) a series of observations on the production of hydrochloric acid, and incidentally on the rate of gastric digestion in suckling infants. In health, the quantity of hydrochloric acid steadily increases from the time milk is taken into the stomach until, in from 1½ to 2 hours after feeding, free acid is present. In from 1½ to 2 hours the stomach becomes empty again, and a little later the acidity begins to decrease, so that the contents of the stomach may even become neutral. In dyspepsia, whether acute or chronic, and in gastro-enteritis, the secretion of acid is slower, and even at the end of two and a-half to three hours there may be no free acid; the digestion of milk is likewise delayed. This observation is in agreement with the fact that acute dyspepsia may be successfully managed by lengthening the intervals between the nursings to three hours, or by diminishing the quantity, which has the effect of leading to the presence of a much greater degree of acidity within the same time. In none of the cases of gastric disease examined was there an excessive acidity. The effect of washing out the stomach before feeding, whether in health or disease, was to increase the quantity of acid secreted during the subsequent digestion, which Wohlmann considers to be an indication for the systematic adoption of this treatment in chronic dyspepsia. He also found that the secretion of acid was slower, and the completion of digestion later in premature children than in those born at full time.

But many other points require consideration, such as the propulsive power of the stomach, the condition of its mucous membrane, &c., and Dr. Bods, writing on the dieting of gastro-intestinal disorders, lays it down that three points must be looked to: 1, the constitutional condition and the state of nutrition of the patient; 2, the surroundings and customary habits of the patient. Thus, the dietetic treatment of the workman must be considered from another standpoint than that of the well to do; 3, the most important point is the prescription of diet with the actual disturbance of digestion in view. The stomach, for example, gets out of order in two of its functions—the motor and the chemical; absorption in the stomach plays a very small part in the functions of the organ, so that an endeavour must be made (by the use of the stomach sound) to discover (1) whether there is a disturbance of the gland function, and whether there are fermentative processes going on; (2) whether the motor activity of the stomach is at fault; or (3) whether both these conditions are present. There are cases, for example, in which the stomach seems incapable, owing to deficiency of gastric juice, of digesting proteids; in these cases the digestion of carbohydrates may be perfect. Proteids in these cases must, therefore, be given in a prepared or semi-digested form. In these cases fat is digested with difficulty, or rather is split up into fatty acids by the fermentation in the duodenum, and so does not enter the lymph channels in the usual form of an emulsion of neutral fat. Sodium chloride is useful in these cases, since it helps to form the hydrochloric acid of the stomach, which tends to stop fermentative processes. On the other hand, there are cases where there is hyperacidity in the stomach. In these cases proteids are exceedingly well digested and carbohydrates but feebly acted upon, so that the digested forms, such as dextrines, malted foods, &c., have to be prescribed. For insufficiency of the motor activity of the stomach, enemata of half a litre, with a proper diet, are beneficial.

A popular misapprehension exists in regard to drinking fluid during meals, it being often thought that the fluid dilutes the gastric juice and so weakens digestion. Mr. Hutchinson refers to this point in a recent number of his "Archives." "I observe with pleasure that the verdict of general experience and common sense has been confirmed by scientific experiment in the matter of taking fluid with meals. Dr. Tev. O Stratievsky, of St. Petersburg, after elaborate trials, has found that fluids materially assist the assimilation of proteids, and announces the following conclusion, which is to be hoped no future experiments will controvert—on the whole, the widely-spread custom of taking fluids during or just before one's meals, proves to be rational and fully justified on strict scientific grounds. To take fluids with the meals is almost as important an adjunct to digestion as is the mastication of solid food preparatory to swallowing it."*

*Refs.: *Brit. Med. Journ. Suppl.*, Oct. 25th, 1890; ibid, Oct. 18th, 1890; July 18th, 1891.

